# How to reach people in the last mile: practical steps

**Published:** 2022-09-20

Establishing a primary eye health care service (see *Community Eye Health Journal* No. 113: Primary eye health care), as close to last mile communities as possible, is an important first step. The primary health care service not only functions as the base for developing outreach services to reach the last mile community, but also links people to the secondary and tertiary eye care services they may need.

Although every country and situation is different, the steps below can help with planning relevant and workable approaches to reaching people in the last mile and supporting them to increase the demand for, and uptake of, services.

## 1. Situation analysis

### a. Define the population

Agree with governments or programme leaders on the criteria for defining last mile populations and/or locations (rural and urban).

### b. Estimate the population size

Estimate the number of people living in the last mile (as defined) or use data from other public health programmes, e.g., polio immunisation or other population-wide health campaigns.

### c. Estimate the need

Find out what proportion of the population in the last mile needs eye care services. For example, in our recent issue on primary eye health care, authors Clare Gilbert and Mapa Prabhath Piyasena estimated that 27% of community members in Asia, 20% of community members in Africa, and 17% of community members in Latin America needed preventive, curative, and rehabilitative eye care services.^4^ These estimates are very general, and will differ significantly between countries and among different groups in the same country. If available, use data from Rapid Assessment of Avoidable Blindness (RAAB) surveys, which produce detailed estimates of eye care need. Data from over 300 RAAB surveys worldwide are available, free of charge, at **www.raab.world**.

Using the known population size in the defined last mile area, and the estimated proportion of people who need care, you can work out the **number of people estimated to need care in the last mile**. This is the **denominator** against which to measure current access and any future progress.

### d. Current access and gaps

If possible, find out how many people in the last mile population have had contact with eye care over the past year. Comparing this to the number of people estimated to need care gives an idea of the number of people who are not being reached at all.

However, simply reaching people is not enough. Referral and follow-up systems need to work well too. For example, try to find out: of those who were reached, what proportion received help such as near vision spectacles or eye drops immediately, and what proportion was referred? Of those who were referred, how many attended, and how many did not? What were the factors responsible?

Just as important is understanding the quality of the clinical care provided, and people's experience of the eye service. Poor outcomes and bad experiences (e.g., long waiting times or being treated as second-class citizens) will harm the reputation of the service and reduce demand and uptake.

### e. Assessment of strengths: what is available in the last mile?

**The community**. Assess the strengths of the last mile community: to what extent are they able to (and do they already) participate in eye care that is offered, and take responsibility for their own health? Are they self-organising – i.e., are there existing structures and organisations in the community that can collaborate with eye care providers to improve access?

**Community systems: health and** **non-health.** Identify formal, informal, and traditional systems and services, and categorise them into helpful/useful and harmful (e.g., traditional practices such as couching).

## 2. Plan intervention strategies

Using the data, focus interventions on who is not being found and who is not attending referral appointments. How can services be adapted to better reach the community? How can people be empowered with the knowledge that they need eye care? How can they be supported to find and make use of the eye care services they need?

Some suggested approaches (in discussion and agreement with the population) are:

- **Shortening the last mile** using technology, mHealth, and telehealth; reducing travel by provider and population, and through specially designed last mile programmes.
- **Addressing cost barriers** through novel payment strategies and insurance schemes, and/or preferential budgets by government
- **Improving the quality of services**, both clinical and non-clinical, by developing special strategies to win trust and confidence in order to improve the demand for, and uptake of, services
- **Building on the strengths of the population in the last mile.**

Cost is an important factor and enough budget must be set aside to build trust and support long-term engagement with communities in the last mile, in order to ensure that communities are involved in a project from inception through to the monitoring and evaluation of services.

## 3. Evaluate, reflect, and make improvements

Continually monitor progress against the denominator (the number of people in the last mile who need eye care) and make changes where needed, in partnership with the community.

“Assess the strengths of the last mile community: to what extent are they able to (and do they already) participate in eye care that is offered, and take responsibility for their own health?”

**Figure:**
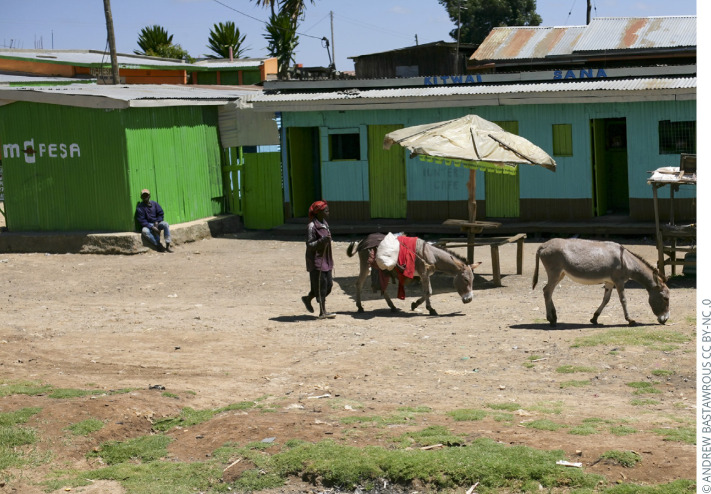
Novel strategies such as cellphone payments can support access to eye care in the last mile. kenya

## 4. Advocate for government investment

Alongside our efforts in eye care, we must also highlight the plight of people in the last mile and advocate for long-term government investment in the provision of basic services in last mile areas. As mentioned earlier, including the last mile in national health care plans is an important step in ensuring buy-in from the highest levels of government, including providing budgetary support. Reaching the last mile is also part of the United Nations target of universal health coverage, which includes minimising the geographical distance between populations and services, and providing essential services of high quality at an affordable cost, so that service recipients are not pushed into poverty or financial hardship. Governments worldwide adopted this target in 2015 and reaffirmed their commitment in 2019, and can therefore be held to account.^5^
